# Identification of novel variants in Iranian consanguineous pedigrees with nonsyndromic hearing loss by next‐generation sequencing

**DOI:** 10.1002/jcla.23544

**Published:** 2020-08-30

**Authors:** Fatemeh Bitarafan, Seyed Yousef Seyedena, Mahdi Mahmoudi, Masoud Garshasbi

**Affiliations:** ^1^ Department of Biology Faculty of Biological Sciences Islamic Azad University, North Tehran Branch Tehran Iran; ^2^ Rheumatology Research Center Tehran University of Medical Sciences Tehran Iran; ^3^ Department of Medical Genetics Faculty of Medical Sciences Tarbiat Modares University Teheran Iran

**Keywords:** autosomal recessive nonsyndromic hearing loss (ARNSHL), Iranian consanguineous pedigrees, next‐generation sequencing (NGS), novel variants

## Abstract

**Background:**

The extremely high genetic heterogeneity of hearing loss due to diverse group of genes encoding proteins required for development, function, and maintenance of the complex auditory system makes the genetic diagnosis of this disease challenging. Up to now, 121 different genes have been identified for nonsyndromic hearing loss (NSHL), of which 76 genes are responsible for the most common forms of NSHL, autosomal recessive nonsyndromic hearing loss (ARNSHL).

**Methods:**

After excluding mutations in the most common ARNSHL gene, *GJB2*, by Sanger sequencing, genetic screening for a panel of genes responsible for hereditary hearing impairment performed in 9 individuals with ARNSHL from unrelated Iranian consanguineous pedigrees.

**Results:**

One compound heterozygote and eight homozygote variants, of which five are novel, were identified: *CDH23:*p.(Glu1970Lys), and p.(Ala1072Asp), *GIPC3*:p.(Asn82Ser), and (p.Thr41Lys), *MYO7A*:p.[Phe456Phe]; p.[Met708Val], and p.(Gly163Arg), *TECTA*:p.(Leu17Leufs*19), *OTOF:*c.1392+1G>A, and *TRIOBP*:p.(Arg1068*). Sanger sequencing confirmed the segregation of the variants with the disease in each family.

**Conclusion:**

Finding more variants and expanding the spectrum of hearing impairment mutations can increase the diagnostic value of molecular testing in the screening of patients and can improve counseling to minimize the risk of having affected children for at risk couples.

## Graphical abstract

In the present study, nine unrelated Iranian consanguineous families with at least one affected individual were tested by next‐generation sequencing (NGS) of 127 known deafness genes. In this report, one compound heterozygote and eight homozygote variants, of which five are novel, were identified: *CDH23:*p.(Glu1970Lys), and p.(Ala1072Asp), *GIPC3*:p.(Asn82Ser), and (p.Thr41Lys), *MYO7A*:p.[Phe456Phe]; p.[Met708Val], and p.(Gly163Arg),*TECTA*:p.(Leu17Leufs*19), *OTOF:*c.1392+1G>A, and *TRIOBP*:p.(Arg1068*). Sanger sequencing confirmed the segregation of the variants with the disease in each family.

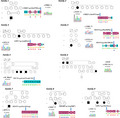

## 1 INTRODUCTION

Hearing loss is the most common sensory disorder in humans. It is estimated that 360 million people worldwide are suffering from hearing loss.^1^ The frequency of congenital deafness ranges from 1 to 2 per 1000 in Western countries, while in Iran it reaches to 1 in 166; in other words, the prevalence of deafness in Iran is estimated to be 2‐3 times higher than the other parts of the world.^2^, ^3^ Iran as one of the consanguinity belt countries, with 38.6% rate of consanguineous marriage which is culturally and socially favored, among the world's most heterogeneous populations, has received a great deal of attention as a potential risk factor for many autosomal recessive disorders including autosomal recessive nonsyndromic hearing loss (ARNSHL).^4^, ^5^

Genetic forms of deafness responsible for more than half of hearing loss cases have been shown to have diverse etiologies, and it is estimated that approximately 1% of all human genes are involved in the biology of hearing.^6^ Congenital hearing loss is the second most common disorder following intellectual impairment in Iran.^3^ Malfunctions of the cochlea and inner ear due to dysfunction of proteins involved in mechanisms related to the adhesion of hair cells, intracellular transport, neurotransmitter release, ionic homeostasis, and cytoskeleton of hair cells can cause hearing impairment. A defect in any part of these mechanisms can cause the disease.^7^ The extremely genetic heterogeneity of deafness can be due to the complexity of the auditory system, which requires coordination of multiple processes controlled by the interaction of various proteins coded by several hundred genes.^6^, ^8^ Up to now, 121 genes have been implicated in the pathogenesis of nonsyndromic deafness in which about 76 genes have been reported to cause ARNSHL (https://hereditaryhearingloss.org/). Causative genes can be classified by their molecular function, homeostasis, hair cell structure, transcription factors, cytokinesis, extracellular matrix, mitochondrial, and other/unknown.^6^, ^9^

Previous studies have shown mutations in *GJB2*, *SLC26A4*, and *TECTA* genes as the most common cause of NSHL in the Iranian population followed by *MYO15A*, *ILDR1, TMC1*, *PJVK, LRTOMT*, *MYO7A*, *OTOF*, and *MARVELD2*.^10^

In spite of tremendous heterogeneity, recently in a cohort of 302 *GJB2*‐negative Iranian probands with ARNSHL, over half of all genetic diagnoses (52%) have been shown to be due to the causative variants in only five genes (*SLC26A4*, *MYO15A*, *MYO7A*, *CDH23*, and *PCDH15*).^1^ In the remaining pedigrees, mutations in 35 other genes including *GIPC3*, *TECTA*, *OTOF*, and *TRIOBP* were identified.^1^

In the present study, 9 unrelated Iranian families with at least one affected individual who were negative for mutations in *GJB2* were screened by next‐generation sequencing (NGS) for 127 known deafness genes. In this report, variants in 6 different genes including three variants in *MYO7A*, two variants in *CDH23* and *GIPC3*, and one variant in *TECTA*, *OTOF*, and *TRIOBP* were identified.

## 2 MATERIALS AND METHODS

### 2.1 Patients and ethics statement

In this study, nine Iranian families with at least one hearing impaired member who was referred to the Department of Medical Genetics, DeNA Laboratory, Tehran, Iran, were investigated. All clinical data of hearing impaired patients in these families were obtained at DeNA Laboratory using a uniform questionnaire according to ACMG guidelines for the etiologic diagnosis of congenital hearing loss, included consanguinity and hearing status of the parents and siblings, age of onset, one or both ears deafness, syndromic or nonsyndromic deafness, presence of accompanying symptoms such as visual anomalies, endocrine abnormalities, thyroid disorders, skin problems, exposure to environmental factors like taking drugs or drinking alcohol during pregnancy, and intrauterine infections.^11^ The hearing impaired individuals in these pedigrees had no obvious vestibular dysfunction, retinal degeneration, or report of other anomalies, suggesting that the families are suffering from nonsyndromic deafness. Evaluation of the deaf patients showed prelingual bilateral nonsyndromic sensorineural hearing loss in all cases. Medical investigations included otoscopy and physical examination by an otolaryngologist and a geneticist. According to audiological evaluations, the severity of deafness varied among patients, ranging from mild to profound (Table 1).

**Table 1:** Clinical details of hearing impaired patients

Family ID	Individual ID	Gender	Hearing impairment					Other clinical features	Family history	Consanguinity
			Onset	Type		Severity^a^	cochlear implants			
Family 1	IV1	Male	Prelingual	Sensorineural	Bilateral	Severe	Yes	No	No	Yes
Family 2	II2	Female	Prelingual	Sensorineural	Bilateral	Severe	No	No	No	Yes
Family 3	II4	Female	Prelingual	Sensorineural	Bilateral	Severe	No	No	No	Yes
Family 4	IV1	Female	Prelingual	Sensorineural	Bilateral	Profound	Yes	No	No	Yes
Family 5	IV6	Male	Prelingual	Sensorineural	Bilateral	Moderate	No	No	Yes	Yes
Family 6	IV5	Female	Prelingual	Sensorineural	Bilateral	Profound	Yes	No	Yes	Yes
Family 7	IV1	Male	Prelingual	Sensorineural	Bilateral	Moderate	Yes	No	No	Yes
Family 8	III1	Female	Prelingual	Sensorineural	Bilateral	Severe to profound	No	No	No	Yes
Family 9	III2	Female	Prelingual	Sensorineural	Bilateral	Severe to profound	No	No	Yes	Yes

^a^ The intensity of hearing loss is classified according to Shearer et al (1999).^45^

**Figure 1:**
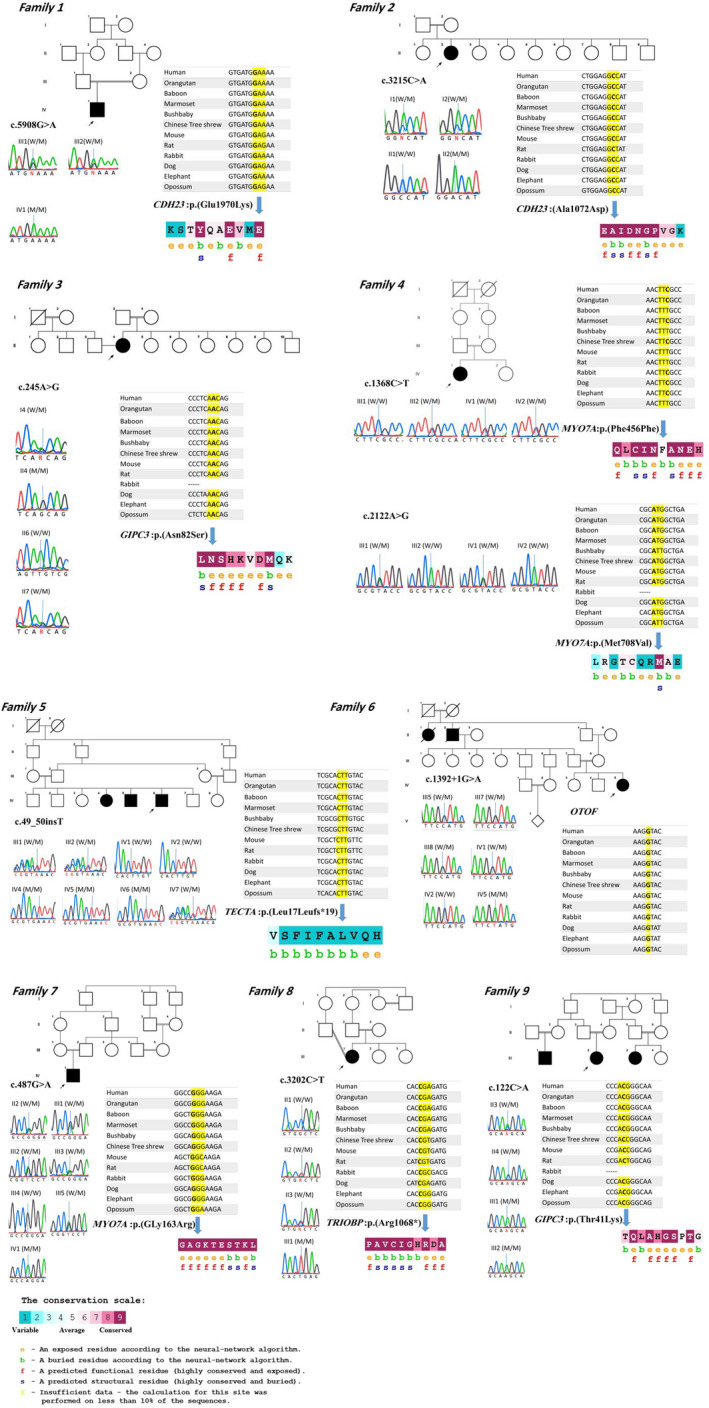
Representative pedigrees, sequence chromatograms confirming the mutations, cross‐species alignments, and ConSurf results of amino acids

In all cases, deaf patients had consanguineous normal parents, suggesting autosomal recessive deafness. Written informed consent for genetic testing was obtained from the adult patients or from their parents in case the patients were under 18 years of age. Some cases were sporadic, while other families had history of multiple affected members with hearing loss. Pedigrees are shown in Figure 1.

### 2.2 DNA extraction

Blood samples were collected from families including 12 patients and 32 normal individuals (Table 2). Genomic DNAs were extracted from the peripheral blood of the patients and all available family members by the High Pure PCR template preparation kit (Roche: Product No. 11814770001).

**Table 2:** List of identified mutations

Family ID	Family member	Age	Affection status	Genotype	Gene	Chromosome location [GRCh37.p13]	Amino acid alternation	Reference
Family 1	IV1	9	Affected	AA	CDH23 NM_022124.6	NC_000010.10:g.73548784C>T Exon43	p.(Glu1970Lys)	This study
	III1	44	Normal hearing	GA				
	III2	40	Normal hearing	GA				
Family 2	II2	21	Affected	AA	CDH23 NM_022124.6	NC_000010.10:g.73468963G>T Exon26	p.(Ala1072Asp)	26
	I1	60	Normal hearing	CA				
	I2	58	Normal hearing	CA				
	II1	30	Normal hearing	CC				
Family 3	II4	34	Affected	GG	GIPC3 NM_133261.3	NC_000019.9:g.3586512T>C Exon2	p.(Asn82Ser)	This study
	I4	60	Normal hearing	AG				
	II6	33	Normal hearing	AA				
	II7	32	Normal hearing	AG				
Family 4	IV1	19	Affected	CT	MYO7A NM_000260.4	NC_000011.9:g.76873190G>A Exon13	p.(Phe456Phe)	This study
	III1	48	Normal hearing	CC				
	III2	38	Normal hearing	CT				
	IV2	14	Normal hearing	CT				
	IV1	19	Affected	AG	MYO7A NM_000260.4	NC_000011.9:g.76886445T>C Exon18	p.(Met708Val)	This study
	III1	48	Normal hearing	AG				
	III2	38	Normal hearing	AA				
	IV2	14	Normal hearing	AA				
Family 5	IV6	32	Affected	InsT/ InsT	TECTA NM_005422.2	NC_000011.9:g120973423_120973424insA (NM_005422.2: c.49_50insT) Exon1	p. (Leu17Leufs*19)	This study
	III1	57	Normal hearing	N/ InsT				
	III2	60	Normal hearing	N/ InsT				
	IV1	44	Normal hearing	N/N				
	IV2	42	Normal hearing	N/N				
	IV4	40	Affected	InsT/ InsT				
	IV5	38	Affected	InsT/ InsT				
	IV7	37	Normal hearing	N/ InsT				
Family 6	IV5	15	Affected	AA	OTOF NM_194248.3	NG_009937.1(NM_194248.3): c.1392+1G>A Intron13	–	39
	III5	60	Normal hearing	GA				
	III7	50	Normal hearing	GA				
	III8	48	Normal hearing	GA				
	IV1	34	Normal hearing	GA				
	IV2	31	Normal hearing	GG				
Family 7	IV1	7	Affected	AA	MYO7A NM_000260.4	NC_000011.9:g.76867722C>T Exon6	p.(Gly163Arg)	31
	II2	50	Normal hearing	GA				
	III1	25	Normal hearing	GA				
	III2	28	Normal hearing	GA				
	III3	31	Normal hearing	GA				
	III4	37	Normal hearing	GG				
	III5	34	Normal hearing	GA				
Family 8	III1	32	Affected	TT	TRIOBP NM_001039141.3	NC_000022.10:g.38121765G>A Exon7	p.(Arg1068*)	34
	II1	38	Normal hearing	CC				
	II2	56	Normal hearing	CT				
	II3	49	Normal hearing	CT				
Family 9	III2	16	Affected	AA	GIPC3 NM_133261.3	NC_000019.9:g. 3585717G>T Exon1	p.(Thr41Lys)	29
	II3	41	Normal hearing	CA				
	II4	44	Normal hearing	CA				
	III1	7	Affected	AA				

### 2.3 Targeted next‐generation sequencing and in silico analysis

All families were negative for mutations in *GJB2*. A custom‐designed NimbleGen chip capturing 127 genes involved in HL based on the deafness variation database (DVD) (http://deafness‐variationdatabase.org/letter) followed by next‐generation sequencing was employed to do genetic screening in proband in each family. List of the genes included in this panel is provided as Table S1. In general, the test examined >95% of the target genes with sensitivity >99%. Point mutations, microinsertion, deletion, and duplication (<20 bp) can be simultaneously detected by this targeted NGS panel. Reads were mapped to the reference human genome (GRCh37, UCSC hg19) using the Burrows‐Wheeler Aligner (http://bio‐bwa.sourceforge.net/). Single‐nucleotide variants (SNVs) and microinsertions‐deletions (indels) were called using SAMtools (http://samtools.sourceforge.net/), based on filtered variants with a mapping quality score of >20, and were annotated using ANNOVAR (http://www.openbioinformatics.org/annovar/). For analysis of the sequencing results, the international publicly available mutation and polymorphism databases such as 1000 Genomes Project (http://www.1000genomes.org/), Exome Aggregation Consortium (ExAC) (http://exac.broadinstitute.org/), Exome Sequencing Project (ESP)(http://evs.gs.washington.edu/EVS/), and Deafness Variation Database (DVD) (http://deafness‐variationdatabase.org/letter) as well as BGI self‐developed local database were employed.^12^ Only variants with a frequency below 1 percent were selected. Previously reported mutations that have been described in Human Gene Mutation Database (HGMD) (http://www.hgmd.cf.ac.uk) and ClinVar (https://www.ncbi.nlm.nih.gov/clinvar) as pathogenic or likely pathogenic were given the highest priority.^13^ Prediction of the consequence of point mutations was obtained from at least three online databases, namely SIFT (https://sift.bii.a‐star.edu.sg/), Polyphen2 (http://genetics.bwh.harvard.edu/pph2/), and MutationTaster (http://www.mutationtaster.org/). In case of intronic variants, Human Splicing Finder (HSF) (http://www.umd.be/HSF3/) which predicts the formation or disruption of splice donor sites, splice acceptor sites, exonic splicing silencer (ESS) sites, and exonic splicing enhancer (ESE) sites was utilized.^14^ For further consideration, the frequency of the variants was checked out on the local database, Iranome (http://www.iranome.ir/). Also, ConSurf (http://www.consurf.tau.ac.il) was applied to check the evolutionary conservation in the region of the mutations (Figure 1).

### 2.4 Segregation analysis

The identified variants were confirmed by direct Sanger sequencing in patients and their all available family members to determine the variants segregation with the disease in these families. Primers surrounding region of the identified variant were designed using Primer3Plus (https://primer3plus.com/cgi‐bin/dev/primer3plus.cgi) web‐based server [PCR conditions and primer sequences are available upon request]. Consequently, DNA sequencing of the PCR products was performed on ABI 3130 with the ABI PRISM BigDye Terminator v. 3.1 sequencing kit (Applied Biosystems, USA). Sequencing chromatograms (Figure 1) were analyzed using CodonCode Aligner software version 8.0.2 (CodonCode Corp).

## 3 RESULTS

This study assessed a total of 9 ARNSHL Iranian families, 9 index cases and their 35 relatives, to confirm the diagnosis of the ARNSHL disease (Table 2). All the patients in this study had consanguineous parents and diagnosed with bilateral congenital sensorineural hearing loss. None of the patients displayed any additional symptoms apart from hearing loss. Targeted NGS of 127 hearing loss‐related genes was carried out in the nine probands. Possible causative variants in each family are summarized in Table 2.

A total of 10 variants in 6 distinct genes (*CDH23*, *GIPC3*, *MYO7A*, *TRIOBP*, *TECTA*, and *OTOF*) in 9 recessive pedigrees (Table 2) were identified. Among them, five variants were previously reported and the other 5 variants were novel. The 10 identified variants included 6 missense, 1 nonsense, 1 intronic, 1 frameshift, and 1 synonymous variant which predicted to affect on splicing by Human Splicing Finder (Table 3).

**Table 3:** Various online databases that used to predict the pathogenicity of the exonic variants

	Family 1	Family 2	Family 3	Family 4		Family 5	Family 6	Family 7	Family 8	Family 9
Gene	*CDH23*	*CDH23 bvn*	*GIPC3*	*MYO7A*	*MYO7A*	*TECTA*	*OTOF*	*MYO7A*	*TRIOBP*	*GIPC3*
Nucleic acid alternation	c.5908G>A	c.3215C>A	c.245A>G	c.1368C>T	c.2122A>G	c.49_50insT	c.1392+1G>A	c.487G>A	c.3202C>T	c.122C>A
Mutation type	Nonsynonymous	Nonsynonymous	Nonsynonymous	Synonymous	Nonsynonymous	Frameshift	Intronic	Nonsynonymous	Stop Gain	Nonsynonymous
Mutation function (ACMG)	Variant of uncertain significance	Variant of uncertain significance	Variant of uncertain significance	Variant of uncertain significance	Variant of uncertain significance	Likely pathogenic	Likely pathogenic	Pathogenic	Pathogenic	Likely pathogenic
SIFT	Damaging	Damaging	Tolerated	–	Damaging	–	–	Damaging	–	Tolerated
Polyphen	Probable damaging	Probable damaging	Probable damaging	–	Benign	–	–	Probable damaging	–	Polymorphism
MutationTaster	Disease causing	Disease causing	Disease causing	Disease causing	Disease causing	Disease causing	Disease causing	Disease causing	Disease causing	Disease causing
1000 Genome	N.R	N.R	N.R	Homozygous: 0 Heterozygous: 1	Homozygous: 0 Heterozygous: 2	N.R	N.R	N.R	N.R	N.R
EXAC all MAF	N.R	N.R	0.000041	0.000223	0.000258	N.R	N.R	N.R	0.000008	N.R
Varsome	Uncertain significance	Uncertain significance	Uncertain significance	Likely benign	Uncertain significance	Pathogenic	–	Pathogenic	Pathogenic	Uncertain significance
Spliceview	–	–	–	–	–	–	Mutant N.R	–	–	–
NetGene2 2.3	–	–	–	–	–	–	Mutant N.R	–	–	–
BDGP	–	–	–	–	–	–	Mutant N.R	–	–	–
HSF	–	–	–	New ESS site ESE site broken						
Segregates in the family	Yes	Yes	Yes	Yes	Yes	Yes	Yes	Yes	Yes	Yes

Abbreviations: ACMG, American College of Medical Genetics and Genomics; ExAC, Exome Aggregation Consortium; MAF, Minor Allele Frequency; NR, not reported.

Sanger sequencing on available family members revealed that these variants segregate with the disease in each family (Table 2 and Figure 1). The in silico pathogenicity predictions for each variant using SIFT, Polyphen2, and MutationTaster software are shown in Table 3.

Family 1: DNA from a 9‐year‐old boy (IV‐1) with cochlear implants due to severe hearing loss was screened for mutations in HL genes by NGS. The sequencing of the total length of 619 167 bp was obtained with a coverage of 98.81%, an average depth of 293.97X, and a minimum depth of 30X. A novel missense variant, c.5908G>A; p.(Glu1970Lys), in exon 43 of *CDH23* was identified. We showed that this variant is segregating with the disease in this family by investigating normal carrier parents (III‐1 and III‐2). This variant has not been reported in 1000 Genome and ExAC databases. Prediction of the consequence of this variant was disease‐causing by mutation tasting, damaging by SIFT, and probable damaging by Polyphen. Based on the ACMG guidelines, the c.5908G>A variant classified as variant of uncertain significance (VUS).

Family 2: DNA from a 21‐year‐old woman (II‐2) with severe hearing loss and no family history was screened for variants in HL genes by NGS. The sequencing of the total length of 620 604 bp was obtained with a coverage of 98.49%, an average depth of 351.7X, and a minimum depth of 30X. A previously reported missense variant, c.3215C>A; p.(Ala1072Asp), in exon 26 of *CDH23* was identified. We showed that this variant is segregating with the disease in this family by investigating her normal parents (I‐1 and I‐2) and her sister (II‐1). This variant has not been reported in 1000 Genome and ExAC databases. Prediction of the consequence of this variant was disease‐causing by mutation tasting, damaging by SIFT, and probable damaging by Polyphen. This variant classified as VUS according to the ACMG recommendations.

Family 3: DNA from a 34‐year‐old woman (II‐4) with severe hearing loss was investigated for variants in HL genes by NGS. The sequencing of the total length of 619 167 bp was obtained with a coverage of 99.01%, an average depth of 354.28X, and a minimum depth of 30X. A novel missense, c.245A>G; p.(Asn82Ser), in exon 2 of the *GIPC3* gene was identified, which was confirmed by Sanger sequencing. We showed that this variant is segregating with the disease in this family by investigating her normal mother (I‐4) and two sisters (II‐6 and II‐7). This variant has not been reported in 1000 Genome database. This variant was predicted to be disease‐causing by mutation tasting and probable damaging by Polyphen. It classified as VUS based on ACMG guidelines.

Family 4: DNA from a 19‐year‐old girl (IV‐1) characterized by profound hearing loss who received cochlear implants was screened for mutations in HL genes by NGS. The sequencing of the total length of 619 167 bp was obtained with a coverage of 99.13%, an average depth of 383.96X, and a minimum depth of 30X. Two novel variants, c.[1368C>T];[c.2122A>G], p.[Phe456Phe];p.[Met708Val] in the *MYO7A* gene, were identified. We investigated these variants in her normal hearing parents (III‐1 and III‐2) and sister (IV‐2) and could show segregation with the disease in this family. Both variants were predicted to be disease‐causing by mutation tasting and based on the ACMG guidelines classified as VUS. According to the HSF the synonymous variant, c.1368C>T predicted to affect on splicing by creation of a new ESS site and disruption of an ESE.

Family 5: DNA from a 32‐year‐old man (IV‐6) suffered from moderate hearing impairment, with two other affected siblings, was screened for mutations in HL genes by NGS. The sequencing of the total length of 619 167 bp was obtained with a coverage of 99.24%, an average depth of 420.44X, and a minimum depth of 30X. A novel insertion, c.49_50insT; p.(Leu17Leufs*19), in exon 1 of the *TECTA* gene was identified. We investigated this variant in her normal parents (III‐1 and III‐2), as well as his three normal (IV‐1, IV‐2 and IV‐7) and two affected siblings (IV‐4 and IV‐5), and therefore could show cosegregation of this variant with the disease in this family. This variant has not been reported in 1000 Genome and ExAC databases. Prediction of the consequence of variant was disease‐causing by mutation tasting. This variant classified based on ACMG guidelines as likely pathogenic.

Family 6: DNA from a 15‐year‐old girl (IV‐5) with profound deafness with cochlear implants was screened for mutations in HL genes by NGS. There was history of other affected individuals in the pedigree. The sequencing of the total length of 620 604 bp was obtained with a coverage of 99.27%, an average depth of 214.02X, and a minimum depth of 30X. A reported splice site variant, c.1392+1G>A, in the *OTOF* gene, was identified. This homozygote variant was absent in her normal parents (III‐7 and III‐8) and her 3 relatives (III‐5, IV‐1, and IV‐2). This variant has not been reported in 1000 Genome and ExAC databases. Prediction of the consequence of variant was disease‐causing by mutation tasting. This variant classified as likely pathogenic based on ACMG guidelines.

Family 7: DNA from a 7‐year‐old boy (IV‐1) with sporadic moderate hearing impairment who has cochlear implants was screened for mutations in HL by NGS. The sequencing of the total length of 620 604 bp was obtained with a coverage of 99.39%, an average depth of 272.86X, and a minimum depth of 30X. A previously reported missense variant, c.487G>A; p.(Gly163Arg), in the *MYO7A* gene, was identified. We showed segregation of this variant with the disease in this family by studying his normal parents (III‐1 and III‐2) and 4 relatives (II‐2, III‐3, III‐4, and III‐5). This variant has not been described in 1000 Genome and ExAC databases. Prediction of the consequence of variant was disease‐causing by mutation tasting, damaging by SIFT, and probable damaging by Polyphen. This variant was classified as pathogenic based on ACMG guidelines.

Family 8: DNA from a 32‐year‐old man (III‐1) with severe to profound bilateral sensorineural hearing impairment with congenital onset was screened for mutations in HL by NGS. There was no family history of deafness in this pedigree. The sequencing of the total length of 620 604 bp was obtained with a coverage of 98.92%, an average depth of 241.61X, and a minimum depth of 30X. A previously reported nonsense variant, c.3202C>T; p.(Arg1068*), in the *TRIOBP* gene, was identified. We showed segregation of this variant with the disease in this family by studying his normal parents (II‐2 and II‐3) and his consanguineous partner (II‐1). This variant has not been reported in 1000 Genome database. Prediction of the consequence of variant was disease‐causing by mutation tasting. This variant classified as pathogenic based on ACMG guidelines.

Family 9: DNA from a 16‐year‐old girl (III‐2) with severe to profound hearing impairment was screened for mutations in HL by NGS. The sequencing of the total length of 620 604 bp was obtained with a coverage of 98.92%, an average depth of 241.61X, and a minimum depth of 30X. A previously reported missense variant, c.122C>A; p.(Thr41Lys), in exon 1 of the *GIPC3* gene, was identified. We showed segregation of this variant with the disease in this family by studying her normal parents (II‐3 and II‐4) and another additional patient in the pedigree (III‐1). This variant has not been reported in 1000 Genome and ExAC databases. Prediction of the consequence of variant was disease‐causing by mutation tasting. This variant classified as likely pathogenic based on ACMG guidelines.

## 4 DISCUSSION

Auditory processing originates in the cochlea of the inner ear, where sounds are detected by sensory hair cells and then transmitted to the central nervous system. The sound waves, after traveling through the external canal and middle ear, lead to the stimulation of hair cells of the organ of Corti by fluids movement inside the cochlea. Each hair cell detects a narrow range of sound frequencies. Information about the sounds including timing, frequency, and intensity is then transmitted through highly efficient ribbon synapses to the spiral ganglion neurons. A defect in any part of this procedure can cause hearing impairment.^15^ There are many genes and loci which are involved in this process. Mutations in genes encoding cytoskeletal proteins, structural proteins, regulatory elements, ion channel, and transport proteins can lead to malfunctions of the cochlea and inner ear.^7^ The congenital hearing loss affects the language and speech development followed by child's education. The early identification of deafness may assist with hearing aid or treatment of the disorder such as cochlear implantation at the earliest possible time which can improve speech and language development.^16^, ^17^

Nonsyndromic hearing loss is the second most common disorder after intellectual disability in Iran, affecting one in 16 individuals. This relatively high incidence of hearing loss may be explained by high consanguinity rate in Iran.^18^ Consanguineous marriage is frequent among Asian, African, and Latin American communities due to various factors such as their tradition, culture, and religion. Large pedigrees are also frequent in these communities.^19^ Consanguineous marriage in Middle Eastern countries is ranging from 20% to 70%. Iran with consanguinity rates of 38% of all marriages, ranging from 15.9% in the northern provinces to 47.0% in the eastern provinces, accounts as one of the countries with high levels of consanguinity.^5^ Single gene autosomal recessive inheritance is responsible for the majority of hereditary hearing loss cases.^19^ Consanguineous matings have long been known as a key etiologic factor in the prevalence of genetic disorders through making disease‐causing recessive genes, inherited from a common ancestor, homozygous. In other words, the probability of inheritaning a similar deleterious recessive allele from both parents increases.^16^

It is confirmed in various reports that the deafness is more common among children of consanguineous marriages. In two epidemiological Saudi Arabian surveys, the prevalence of SNHL has been shown to be 66% and 36.6%, respectively, out of which about 45% and 47% of the children had consanguineous parents.^19^ In an Indian case‐control study, the rates of affected children with consanguineous and nonrelated parents have been shown to be 48% and 28%, respectively.^17^ Parental consanguinity was shown to be more common in Qatari families with hearing impaired patients compared to ones with normal hearing children, 60.5% versus 25.3%.^20^ In a large‐scale study in Oman, it was found that 70% of the hearing impaired children had blood relative parents.^21^ The parental consanguinity rate of hearing impaired patients was measured to be over 60% in several reports from Iran.^22^, ^23^, ^24^, ^25^

Nowadays with the advent of NGS, identification of molecular defects involved in HL has been accelerated.

We have studied nine Iranian families, comprising at least one affected individual with nonsyndromic bilateral autosomal recessive prelingual hearing loss. We assessed a total of 9 index cases and their 35 relatives, to confirm the diagnosis of the ARNSHL disease. All deaf probands were born to consanguineous parents. Targeted NGS of 127 hearing loss‐related genes was carried out in the 9 probands, which allowed us to detect 5 reported and 5 novel variants in 6 distinct deafness genes including *CDH23*, *GIPC3*, *MYO7A*, *TRIOBP*, *TECTA*, and *OTOF*.

A novel missense variant, c.5908G>A; p.(Glu1970Lys), in exon 43 and a previously reported missense mutation,^26^ c.3215C>A; p.(Ala1072Asp), in exon 26 of *CDH23* were identified in families 1 and 2, respectively. *CDH23* encodes a putative calcium‐dependent adhesion molecule protein with 27 extracellular cadherin (EC) domains, a single transmembrane domain, and a short cytoplasmic domain. Cadherin 23 is required for proper morphogenesis of hair bundles of inner ear neurosensory cells.^27^ Previous studies have revealed the importance of ethnic diversity of genetic variants in *CDH23*. Mutations in *CDH23* are one of the most important pathogenic causes of autosomal recessive nonsyndromic hearing loss (DFNB12) in Iranian populations.^1^ Mutations in the *CDH23* gene are known to cause both Usher syndrome type 1D (USH1D) and nonsyndromic hearing loss (DFNB12). To date, at least 80 pathogenic variants of the *CDH23* have been reported in familial or sporadic patients of USH1D and DFNB12 worldwide. Usually, pathogenic missense mutations in any domain of the protein can lead to DFNB12, whereas nonsense, splice site, and frameshift mutations can cause USH1D.^28^ In this study, two missense homozygous variants, p.(Glu1970Lys) and p.(Ala1072Asp), in *CDH23* affecting two highly conserved residues in the extracellular domains of EC19 and EC10, respectively, were detected (https://www.uniprot.org/).

A novel missense variant, c.245A>G; p.(Asn82Ser), in exon 2 and a previously reported missense mutation,^29^ c.122C>A; p.(Thr41Lys), in exon 1 of the *GIPC3* gene were identified in families 3 and 9, respectively. *GIPC3* encodes a 312 amino acid protein that contains 3 domains: an N‐terminal GIPC homology domain (GH1), a central PDZ domain, and a C‐terminal GH2 domain. GIPC3 localizes to inner ear sensory hair cells and is important in peripheral auditory signal transmission.^29^ The GH1, PDZ, and GH2 domains are well conserved among GIPC1, GIPC2, and GIPC3 orthologs. GIPC proteins are involved in the trafficking, signaling, and recycling of various transmembrane proteins. They regulate a variety of cellular processes including proliferation, planar cell polarity, cytokinesis, and migration. Dysregulation of GIPCs results in human pathologies, such as hearing loss and cancer.^30^ The two homozygote variants identified in this study, p.(Asn82Ser) and p.(Thr41Lys), are affecting the GH1 domain.^29^

Two novel variants, c.1368C>T; p.(Phe456Phe) and c.2122A>G; p.(Met708Val) in compound heterozygote state in family 4 and a previously reported missense mutation,^31^ c.487G>A; p.(Gly163Arg), in family 7 were identified in the *MYO7A* gene. *MYO7A* encodes the actin‐binding motor protein, which is involved in differentiation, morphogenesis, and organization of cochlear hair cell bundles.^32^ The myosin‐VIIa protein contains different domains including a myosin head‐like domain, which contains the crucial ATP‐binding site and actin‐bind site, five IQ motifs, a coiled‐coil region, two MyTH4 domains, two FERM domains, and a SH3 domain. Mutations in the *MYO7A* gene have been identified to be associated with nonsyndromic hearing loss (DFNB2, DFNA11) and Usher syndrome type 1B (USH1B).^33^ Here, we identified compound heterozygous missense variants *MYO7A*:p.[Phe456Phe]; p.[Met708Val] and a homozygous variant, p.(Gly163Arg), in two Iranian families with nonsyndromic hearing loss which are affecting the myosin head‐like domain containing residues 1 to 729 of the protein. The synonymous variant; c.1368C>T; p.(Phe456Phe) predicted by HSF to affect splicing by creation of a new ESS site and disruption of an ESE. The p.(Gly163Arg) variant also affects the ATP‐binding site (158‐165).^33^

A previously reported nonsense variant,^34^ c.3202C>T; p.(Arg1068*), in exon 7 of the *TRIOBP* gene was identified in family 8. *TRIOBP* encodes a filamentous actin‐binding protein that has been identified as the gene for DFNB28 deafness. *TRIOBP* variants are not a common cause of HL. To date, over 30 point mutations have been reported in the *TRIOBP* gene. Previous studies have suggested exon 7 of *TRIOBP* as a hotspot for mutations, probably due to presence of repetitive sequences.^32^, ^34^ The protein contains two types of domains, N‐terminal pleckstrin homology (PH) and C‐terminal coiled‐coil. Studies have revealed that *TRIOBP* directly binds and stabilizes the F‐actin structures, presumably via their nonconventional actin‐binding sites.^34^ TRIOBP protein has multiple roles in the organization of actin‐cytoskeleton, proper centrosomal localization and segregation of chromosomes during cell division, and cell cycle regulation.^34^

The proband and his affected siblings in family 5 carried a novel likely pathogenic insertion, c.49_50insT; p.(Leu17Leufs*19), in exon 1 of the *TECTA* gene which was absent in the unaffected members of the family. *TECTA* gene, located on 11q22‐q24, has been implicated both in autosomal dominant (DFNA) and autosomal recessive (DFNB) forms of nonsyndromic hearing loss. The gene comprises 23 exons which encodes one of the major noncollagenous glycoproteins of the tectorial membrane, alpha‐tectorin. The protein is a part of the noncellular matrix which lies over the stereocilia of the cochlear hair cells and is critical both for the mechanical amplification of sound and its transmission to the inner hair cells. Mutations in various parts of alpha‐tectorin lead to deafness at different frequencies. Studies on different populations have shown that alpha‐tectorin is among the top 10 genes responsible for ARNSHL. Previous studies have shown that *TECTA* mutations account for about 4.13% of ARNSHL among *GJB2* negative Iranian families.^35^, ^36^ *TECTA* is associated with a moderate‐to‐severe audio profile.^35^ Mainly missense mutations of *TECTA* cause ADNSHL (DFNA8/12), while the majority of autosomal recessive NSHL (DFNB21) variants are truncating and most likely loss‐of‐function mutations.^36^, ^37^ The variant reported in this study is an insertion, c.49_50insT; p.(Leu17Leufs*19), within the signal peptide of alpha‐tectorin (https://www.uniprot.org/uniprot/O75443). Mutations in signal peptides can affect directing of proteins to their proper cellular and extracellular locations and the translocation of proteins across the cytoplasmic membrane.^38^

The c.1392+1G>A variant in the *OTOF* gene, which we found in the proband of family 6, has been recently reported as a pathogenic splice site variant in another Iranian family.^39^ This variant is affecting the donor splice site of intron 13, in which G nucleotide is replaced by A. Splice site software tools and MutationTaster, Table 3, predicted that this variant causes lose of donor splice site and leading to intron retention.^38^ Previous studies have shown that a single amino acid change, even in nonconserved residues, in 1 C2 domain severely affects protein stability and localization. This could explain the profound deafness phenotype due to the severe effect of the splice site variant on C2C and downstream domains of the protein.^39^ The *OTOF* gene (DFNB9) is mainly expressed in cochlear inner hair cells and is necessary for synaptic exocytosis at the auditory ribbon synapse. Because of the expected good outcomes of cochlear implantation for patients with *OTOF* mutations, it is important to perform mutation screening for *OTOF* to select the appropriate intervention.^40^ To date, more than 100 pathogenic variants including missense, nonsense, frameshift, splice site, deletion, and duplication have been found in various populations, nearly a third of which are from the Middle East, especially Pakistan and Turkey. Studies in Iran suggested that ARNSHL due to *OTOF* gene mutations ranges from 0.7% to 2.6%. There are few reports of splice site mutations.^39^ Hearing loss due to *OTOF* mutations is characterized by abnormal inner hair cell function and dyssynchrony of neural transmission of the auditory signal from the inner ear to the auditory nerve and brainstem.^41^ The *OTOF* gene, located on chromosome 2p23.1, encodes a membrane‐anchored cytosolic protein, otoferlin, with several isoforms.^42^, ^43^ It is believed that variants affecting the long isoform of this gene cause ARNSHL.^41^ The long isoform, which is thought to be required for normal hearing, involves 48 coding exons which contains six C2 domains (C2A‐C2F) and a transmembrane domain (TM).^43^, ^44^ Otoferlin plays a role in the calcium‐dependent fusion of vesicles to the plasma membrane.^43^

## 5 CONCLUSION

In this study, a total of 10 variants in the patients were identified. Among them, five mutations were previously reported and the other five variants were novel. Accurate identification of causative mutations plays a key role in affected families to offer them preimplantation genetic diagnosis (PGD), prenatal diagnosis (PND), or further therapy strategies. Besides finding more mutations and new genes provides the possibility to do further studies on the pathophysiology of this disease and identify the involved pathways and mechanisms.

## AUTHORS' CONTRIBUTIONS

MG and FB conceived and designed the experiments. MG and F.B contributed to data collection. FB and MG wrote the paper. MG, SY. S, and M. M supervised the work. All authors read and approved the final manuscript.

## Supporting information

Tab S1
