# Supplementary material for: Identification of novel variants in Iranian consanguineous pedigrees with nonsyndromic hearing loss by next‐generation sequencing
Source: J Clin Lab Anal. 2020 Aug 30;34(12):e23544. doi: 10.1002/jcla.23544 (PMC7755797; doi:10.1002/jcla.23544)
Supplement: Supplementary file 1 — Tab S1 [file JCLA-34-e23544-s001.docx]

| Table S1 (Test information ) | |
| --- | --- |
| Disease name | Gene tested |
| Autosomal recessive nonsyndromic hearing impairment | *GJB2*; *GJB6*; *MYO7A*; *MYO15A*; *FOXI1*; *KCNJ10*; *SCL26A4*; *TMIE*; *TMC1*; *TMPRSS3*; *OTOF*; *CDH23*; *ATP2B2*; *GIPC3*; *STRC*; *OTOG*; *USH1C*; *TECTA*; *OTOA; PCDH15*; *RDX*; *GRXCR1*; *TRIOBP*; *CLDN14*; *MYO3A; WHRN*; *ESRRB*; *ESPN*; *MYO6*; *GJA1*; *HGF*; *ILDR1; MARVELD2*; *DFNB59*; *SLC26A5*; *LRTOMT*; *LHFPL5; BSND*; *MSRB3*; *LOXHD1*; *TPRN*; *GPSM2*; *PTPRQ; SERPINB6*; *GJB3* |
| Autosomal dominant nonsyndromic hearing impairment | *ACTG1*; *CCDC50*; *CEACAM16*; *COCH*; *CRYM*; *DFNA5*; *DIABLO; DIAPH1*; *DSPP*; *EYA4*; *GJB2*; *GJB3*; *GJB6*; *GRHL2*; *KCNQ4*; *MIR96*; *MYH14*; *MYH9*; *MYO1A*; *MYO6*; *MYO7A*; *POU4F3*; *SIX1*; *SLC17A8*; *TECTA*; *TJP2*; *TMC1*; *WFS1*; *DIAPH3* |
| X-link hereditary hearing impairment | *PRPS1*; *POU3F4*; *SMPX* |
| Maternally inherited hearing impairment | *MT-RNR1; MT-TS1* |
| Syndromic hearing impairment | *SERAC1; PDSS1; FGFR3; FGFR1; FGFR2; PHEX; DLX5; TNFRSF11B; COL2A1; COL11A1; COL9A1; COL9A2; COL4A3; COL4A4; COL4A5; BSND; SOX9; PAX2; GATA3; SLC19A2; IGF1; PAX3; MITF; SNAI2; EDNRB; EDN3; SOX10; HOXA1; SOBP; EYA1; SIX5; SIX1; CHD7; SEMA3E; SMAD4; FGF3; TCOF1; PRRX1; GLI3; HOXA2; KCNQ1; KCNE1; CACNA1D; ALMS1; LRP2; TIMM8A; NDP; WFS1; OPA1; SLC4A11; MYO7A; USH1C; CDH23; PCDH15; USH1G; USH2A; ADGRV1; PDZD7; WHRN; CLRN1; MT-TK; MT-TE; MT-TL1; SLC26A4; KCNJ10; FOXI1* |
